# Influence of Y_2_O_3_ Particle Size on the Microstructure, Corrosion Resistance, and Wear Resistance of Electrodeposited Ni-W-Y_2_O_3_ Composite Coatings

**DOI:** 10.3390/ma19132850

**Published:** 2026-07-03

**Authors:** Shilong Xing, Shuo Wu, Zhikun Li, Xiaocong Li, Wenbo Li, Chuanhai Jiang, Chengxi Wang

**Affiliations:** 1 School of Construction Machinery, Shandong Jiaotong University, 5001 Haitang Road, Jinan 250357, China; xsl0404@sdjtu.edu.cn; 2 School of Materials Science and Engineering, Shanghai Jiaotong University, 800 Dongchuan Road, Shanghai 200240, China; 3 School of Navigation and Shipping, Shandong Jiaotong University, Weihai 264209, China; lizhikun0321@163.com (Z.L.);; 4 Faculty of Transportation Engineering, Kunming University of Science and Technology, Kunming 650500, China; sjtucxw@163.com

**Keywords:** electrodeposition, Ni-W-Y_2_O_3_ composite coating, micro/nano Y_2_O_3_ particles, corrosion resistance, wear resistance

## Abstract

**Highlights:**

- Micro/nano Y_2_O_3_ particle incorporation enhances microstructure optimization and refines the crystallite size of the composite coatings.
- Nano-sized Y_2_O_3_ particles significantly improve the corrosion resistance of the composite coatings in acidic and neutral environments, whereas micro-sized Y_2_O_3_ particles have minimal impact on corrosion performance.
- Both micro-sized and nano-sized Y_2_O_3_ particles effectively reduce the coefficient of friction and improve the wear resistance of the composite coatings, with nano particles demonstrating superior performance.
- The mechanisms through which micro/nano Y_2_O_3_ particles optimize the microstructure and significantly enhance the wear resistance of composite coatings are elucidated.

**Abstract:**

Ni-W-Y_2_O_3_ composite coatings were electrodeposited from a sulfate citrate electrolyte incorporating Y_2_O_3_ particles of three different sizes: 50 nm, 1 μm, and 2 μm. This study systematically investigates the impact of Y_2_O_3_ particle size on the microstructure, microhardness, corrosion resistance, and wear resistance of the composite coatings. The incorporation of Y_2_O_3_ particles leads to notable grain refinement, with the crystallite size decreasing from 31.8 nm for the pure Ni W coating to 17.3 nm, 19.0 nm, and 20.1 nm for the C-50 nm, C-1 μm, and C-2 μm coatings, respectively. Correspondingly, the microhardness increases from 540.2 HV for the unreinforced coating to 732.5 HV, 641.0 HV, and 629.5 HV for the C-50 nm, C-1 μm, and C-2 μm coatings, respectively. In terms of corrosion resistance, the C-50 nm coating exhibits the best performance in both acidic and neutral media, with the lowest corrosion current density of 3.72 μA/cm^2^ in 10 wt.% H_2_SO_4_ and 3.53 μA/cm^2^ in 3.5 wt.% NaCl. In contrast, micron-sized particles show limited improvement in acidic media and even degrade the corrosion resistance in neutral NaCl solution, where the corrosion current density increases to 7.68 μA/cm^2^ and 7.74 μA/cm^2^ for the C-1 μm and C-2 μm coatings, respectively. The wear resistance of the composite coatings is significantly enhanced by the incorporation of Y_2_O_3_ particles, with the wear rate decreasing from 17.75 × 10^−6^ mm^3^ N^−1^ m^−1^ for the pure Ni-W coating to 5.65 × 10^−6^ mm^3^ N^−1^ m^−1^ for the C-50 nm coating. The presence of Y_2_O_3_ particles transitions the wear mechanism from predominantly adhesive wear to a combination of adhesive and abrasive wear. These results demonstrate that the particle size of Y_2_O_3_ plays a critical role in determining the microstructure and overall performance of Ni-W-Y_2_O_3_ composite coatings, with nano-sized particles offering the most significant improvements.

## 1. Introduction

Chromium-plated coatings are widely used to enhance the surface properties of metal parts due to their excellent corrosion and wear resistance [1]. However, the electrolytes employed in electroplating contain toxic hexavalent chromium ions, posing risks to production safety and environmental health. Hexavalent chromium is classified as a Group 1 carcinogen, with a permissible exposure limit of 5 μg/m^3^ as an 8-h time-weighted average established by the U.S. Environmental Protection Agency. These severe health and environmental concerns have motivated the search for alternative coating materials, among which nickel-matrix composite coatings have emerged as promising candidates. Nickel-matrix composite coatings have emerged as viable alternatives to hard chromium coatings [2,3,4]. Electrodeposited nickel-tungsten (Ni-W) coatings are not only cost-effective and environmentally benign but also exhibit superior corrosion and wear resistance compared to traditional electroplated hard chromium coatings [5,6]. Consequently, Ni-W alloy coatings have garnered significant attention for applications in electrocatalysis, electrolysis, anti-corrosion coatings, wear-resistant coatings, microelectronics, and machinery [7].

The incorporation of reinforced particles into Ni-W alloy coatings can lead to significant microstructural evolution, resulting in enhanced corrosion resistance, oxidation resistance, and wear resistance. Various types of particles, including SiC [8,9,10], TaC [7], ZrO_2_ [11,12,13,14,15], TiN [16], CeO_2_ [17,18,19], TiB_2_ [20], and Al_2_O_3_ [21,22,23], have been successfully co-deposited with Ni-W alloys, each contributing to the improvement of specific coating properties. Das et al. [24] demonstrated that incorporating micro-sized diamond particles significantly increased the hardness and wear resistance of Ni-W/diamond composite coatings. Yari et al. [25] investigated the effects of Al_2_O_3_ nanoparticles on the microstructure and morphology of Ni-W coatings, revealing that the presence of these nanoparticles influenced the coating’s grain size, tungsten content, and metal deposition rate. Sangeetha et al. [26] explored Ni-W/BN(h) composite coatings and showed that variations in BN(h) concentration altered the structure and properties of the coatings. Similarly, Li et al. [27] found that adding nano-BN particles enhanced the microhardness, wear resistance, and corrosion resistance of Ni-W/BN(h) composite coatings. Wang et al. [28] prepared Ni-W-SiO_2_ nanocomposite coatings, noting that nano-SiO_2_ particles markedly affected the microstructure, composition, and corrosion resistance of the coatings. Yao et al. [29] developed Ni-W-Si_3_N_4_ composite layers and observed that the best electrochemical corrosion resistance is achieved at 5.0 g/L, attributed to the blocking of anodic current pathways by Si_3_N_4_ particles, increased grain boundaries promoting passive film nucleation.

Rare earth oxides, such as CeO_2_ [30], La_2_O_3_, and Y_2_O_3_, are effective reinforcing agents widely utilized in the design and development of composite coatings. Notably, Y_2_O_3_ is pivotal in enhancing the performance of the composite coatings due to its superior ability to mitigate microcracks, increase hardness, and improve both corrosion and wear resistance. Zhang et al. [31] demonstrated that Y_2_O_3_ significantly improves the formation quality of composite coatings by affecting the distribution of Ni and Cr elements within the coating. Wang et al. [32] examined the impact of micro-sized Y_2_O_3_ particles on the microstructure and wear properties of Ti-6Al-4V coatings, finding that coatings with optimal Y_2_O_3_ particle content exhibited the highest hardness and wear resistance. Yue et al. [33] further showed that the incorporation of Y_2_O_3_ notably enhanced the microhardness and wear resistance of the composite coating, shifting the wear mechanism to minor peeling.

Despite extensive research on particle-reinforced composite coatings of various sizes and types, there is a notable scarcity of comparative studies examining how different sizes of the same particle affect the performance of these coatings. While various particle-reinforced Ni-W composite coatings have been extensively investigated, most previous studies have focused on a single particle size or have compared particles of different chemical compositions. A systematic comparative study examining how the size of the same reinforcing particle influences the microstructure and properties of Ni-W composite coatings has been notably lacking. The present study addresses this gap by employing Y_2_O_3_ particles of three distinct sizes, namely 50 nm, 1 μm, and 2 μm, while keeping all other deposition parameters constant, thereby enabling a direct and unambiguous assessment of the particle size effect.

More importantly, contrary to the general expectation that the incorporation of hard particles monotonically improves coating performance, our results reveal that the effect of Y_2_O_3_ particle size is highly property-dependent and environment-sensitive. In particular, micron-sized Y_2_O_3_ particles are found to degrade the corrosion resistance of the composite coating in neutral NaCl solution, a phenomenon that has not been previously reported for Y_2_O_3_-reinforced Ni-W coatings. This unexpected finding challenges the simplistic assumption that particle incorporation is always beneficial and underscores the critical importance of particle size selection in composite coating design. To accomplish this, a series of composite coatings was fabricated using Y_2_O_3_ particles in both nano and micron sizes. The resulting coatings were evaluated for a range of properties, including microstructural characteristics, microhardness, wear resistance, and corrosion resistance.

## 2. Experimental Section

### 2.1. Coating Preparation

Ni-W-Y_2_O_3_ composite coatings reinforced with various Y_2_O_3_ particle sizes were synthesized using direct current electrodeposition from a sulfate-citrate electrolyte. The electroplating solution comprised deionized water and specific analytical reagents, detailed in Table 1. The particle sizes of the as-received Y_2_O_3_ powders were determined by statistical analysis of SEM images using Nano Measurer software V1.2.0.5 (Department of Chemistry, Fudan University), with more than 100 particles counted for each sample to ensure statistical relevance. The average particle sizes were found to be 56.6 nm, 1.3 μm, and 2.0 μm for the nominally 50 nm, 1 μm, and 2 μm powders, respectively. Y_2_O_3_ particles of different sizes purchased from Shanghai Macklin Biochemical Co. (Shanghai, China) were added to the electrolyte at a concentration of 10 g/L, ensuring particle size was the sole variable. Prior to electroplating, a 30-min ultrasonic treatment was employed to ensure complete dispersion of the particles in the solution. The resulting Ni-W-Y_2_O_3_ composite coatings are denoted as C-50 nm, C-1 μm, and C-2 μm, corresponding to the Y_2_O_3_ particle sizes, with the Ni-W alloy coating labeled as C0 for comparison. A pure nickel sheet (15 cm × 15 cm) served as the anode, and a stainless-steel plate of the same dimensions was used as the cathode. The stainless-steel cathode was activated in a 10 vol% HCl solution for 30 s and subsequently cleaned with deionized water. The anode and cathode were positioned vertically in the electrolyte at a constant separation of 30 mm. The electroplating solution was maintained at 70 ± 1 °C using a water bath, and the pH was kept at 8.0 ± 0.2, adjusted with 10 vol% NaOH and 10 vol% HCl solutions. Magnetic stirring of the electrolyte was performed at 300 rpm throughout the deposition process. Post-electrodeposition, samples underwent ultrasonic treatment in deionized water for 5 min to remove loosely bound particles from the coating surface.

### 2.2. Characterization

#### 2.2.1. Scanning Electron Microscopy

The surface morphology and chemical composition of the composite coatings were analyzed using a Scanning Electron Microscope (SEM, JSM-7800F, Tokyo, Japan) and Energy Dispersive X-ray Spectroscopy (EDS, JSM-7800F, Tokyo, Japan), respectively.

#### 2.2.2. Transmission Electron Microscopy and X-Ray Diffraction

The microstructure and grain distribution were examined with a Transmission Electron Microscope (TEM, JEOL-2100F, Tokyo, Japan). Grain size measurements were performed using DigitalMicrograph software V 1.2.44. The process involves thresholding the source image, identifying the grains based on this threshold, and applying circular approximation to determine the diameters of the contour grains. X-ray Diffraction (XRD) patterns were recorded using a Rigaku Ultima IV diffractometer (Rigaku Corporation, Akishima, Japan), equipped with a Cu target and a D/tex one-dimensional high-speed detector, operating at a scanning rate of 2° min^−1^ and a step size of 0.02°. The crystallite size and microstrain of the Ni-W-particles composite coatings were determined from the (111) peak using the Voigt method and the Scherrer equation [34,35]:

(1) $$\beta_{C}^{h}=\beta_{C}^{f}+\beta_{C}^{g}\;;\;\beta_{G}^{h^{2}}=\beta_{G}^{f^{2}}+\beta_{G}^{g^{2}}$$

(2) $$D=\frac{\lambda }{\beta_{C}^{f}\;cos\theta };\;\varepsilon =\frac{\beta_{G}^{f}}{4\tan\theta }$$

where *β* represents integral breadth, *h*, *f*, and *g* represent the measured, structurally broadened, and standard line profiles, respectively, *D* is the crystallite size, and *ε* is the microstrain. *C* and *G* represent the Cauchy and Gaussian components, respectively. *λ* and *θ* represent the wavelength of Cu-Ka and the Bragg angle, respectively. The Voigt single-peak method used in this study is widely accepted for crystallite size and microstrain analysis of nanocrystalline coatings, as it effectively separates the contributions of size and strain to peak broadening.

#### 2.2.3. Electrochemical Corrosion Testing

The corrosion resistance of the composite coatings was assessed using potentiodynamic polarization and electrochemical impedance spectroscopy (EIS) in 3.5 wt.% NaCl and 10 wt.% H_2_SO_4_ solutions at room temperature. A standard three-electrode electrochemical cell was employed, with a saturated calomel electrode as the reference, a platinum sheet as the auxiliary electrode, and the as-deposited coatings (with a 10 mm diameter circular exposed area) as the working electrodes. Potentiodynamic polarization was performed at a scan rate of 1 mV/s, starting from an initial potential of −800 mV. EIS measurements were conducted at the open-circuit potential (E_ocp_) over a frequency range of 0.01 Hz to 100 kHz, with a 5 mV sinusoidal perturbation. All electrochemical measurements were performed on at least three independently prepared samples, and the results showed good reproducibility. These experiments elucidated the corrosion behavior of the composite coatings in various aggressive chemical environments.

#### 2.2.4. Microhardness and Tribological Testing

The microhardness of the composite coating was evaluated using a Vickers microhardness tester (DHV-1000, Caikon, Shanghai, China) with a load of 0.98 N applied for 15 s. The average microhardness was determined from five random measurements. The C0 coating exhibits an average thickness of approximately 26.85 μm, with a deposition rate of about 0.45 μm/min. The composite coatings are expected to have comparable thicknesses, as they were deposited under identical conditions. The indentation depth under this load was less than one-tenth of the coating thickness, ensuring that the measured hardness reflects only the coating’s properties without interference from the underlying substrate. Wear and friction characteristics of the composite coatings were assessed using a tribometer (MFT-5000, Anton Paar, Shanghai, China) with a GCr15 steel counterbody ball of 6 mm diameter under ambient conditions at room temperature. The GCr15 steel has a nominal composition of 0.95–1.05 wt.% C, 0.15–0.35 wt.% Si, 0.25–0.45 wt.% Mn, 1.30–1.65 wt.% Cr, and balanced Fe. The tests were conducted with linear reciprocating motion, an applied load of 4 N, a reciprocating frequency of 5 Hz, an amplitude of 5 mm, and a sliding duration of 600 s. This duration was selected based on preliminary tests, which confirmed that it was sufficient to establish a steady-state wear regime and produce clearly measurable wear tracks on all coating samples. The wear volume was determined from the cross-sectional profile of the wear tracks obtained by profilometry. The cross-sectional area was integrated from the profile depth across the track width, and the wear volume was calculated by multiplying this area by the total sliding distance. The average wear rate was calculated using the formula [36]:

*w* = *dV*/(*dF*·*dL*) (3)

where *w* is the specific wear rate, *V* is the wear volume, *F* is the applied load, and *L* is the sliding distance. Three replicate tests were performed under identical conditions to ensure accuracy and obtain average values.

### 2.3. Statistical Analysis

All experiments were performed on at least three independently prepared samples for each coating formulation to ensure reproducibility. The results are reported as mean values with corresponding standard deviations. For crystallite size and microstrain measurements, the values were determined from the (111) diffraction peak using the Voigt single-peak method on three independent coatings. For wear testing, friction coefficient and wear rate data were obtained from three replicate tests performed under identical conditions. The standard deviations were calculated using Microsoft Excel, and the error bars in the figures represent the standard deviations from the independent measurements. No additional statistical significance tests were performed, as the observed differences between coating groups were sufficiently large and consistent across replicates to support the conclusions drawn in this study.

## 3. Results and Discussion

### 3.1. Characterization of Y_2_O_3_ Particles

**Figure 1:**
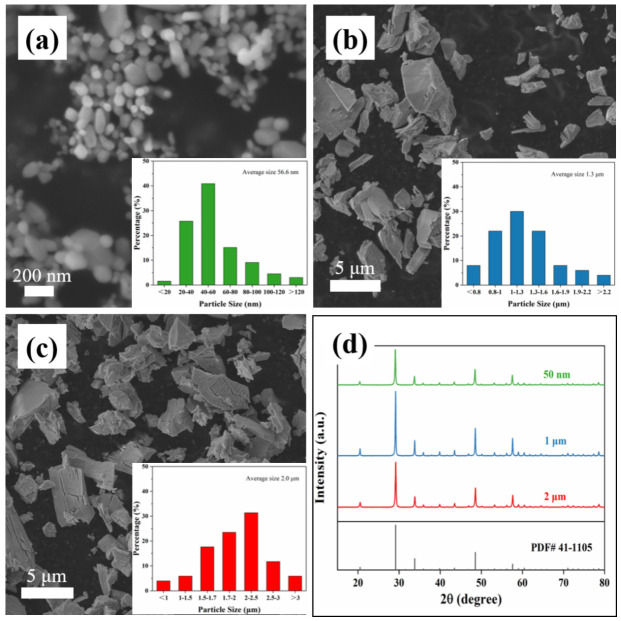
SEM morphologies and histogram of particle size distribution of Y_2_O_3_ particles, (**a**) 50 nm, (**b**) 1 μm, (**c**) 2 μm, along with (**d**) the corresponding XRD patterns.

Figure 1 displays the morphologies and XRD patterns of various Y_2_O_3_ particles. All Y_2_O_3_ particles exhibit irregular, multi-faceted shapes. The average sizes of the three types of Y_2_O_3_ particles were determined to be 56.6 nm, 1.3 μm, and 2.0 μm, respectively. Additionally, the XRD patterns in Figure 1d demonstrate that the diffraction spectra for all particle types align closely with standard PDF card (No. 00-041-1105) data.

### 3.2. Surface Morphologies and Compositions

**Figure 2:**
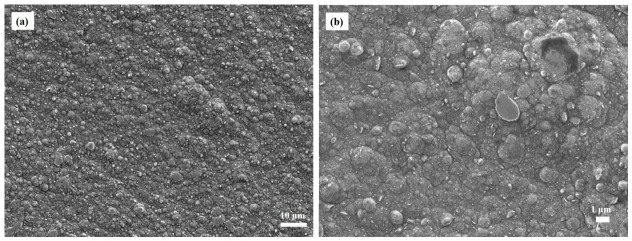
Top-view SEM images of C0 coating (**a**) 1000× and (**b**) 5000×.

**Figure 3:**
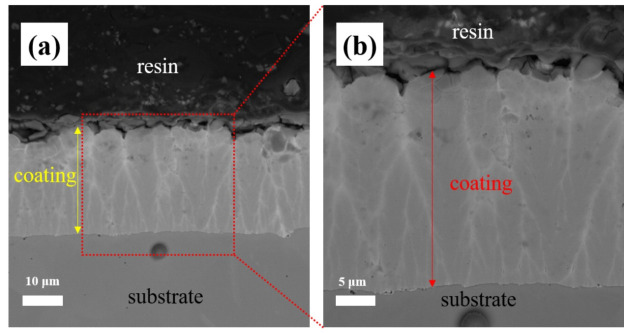
(**a**) Cross-sectional SEM images of C0 coating and (**b**) magnification.

Figure 2 illustrates the surface morphology of the Ni-W alloy coating (C0), which exhibits a cauliflower-like structure characterized by a dense, pore-free surface with no visible cracks. The large interface energy between the substrate and the coating promotes the growth of three-dimensional crystal nuclei, resulting in columnar structures and the observed cauliflower-like morphology [37]. Figure 3 depicts the cross-sectional view of the C0 coating, revealing an average thickness of 26.85 μm. The deposition rate for the C0 coating is approximately 0.45 μm/min. The coating is firmly adhered to the substrate, featuring a dense internal structure and pronounced surface undulations, which are attributed to the cauliflower-like surface morphology of the Ni-W alloy coating.

The electrodeposition of Ni-W-Y_2_O_3_ composite coatings follows a three-stage growth model. In the initial stage, nucleation occurs predominantly on the substrate, following instantaneous nucleation behavior typical of diffusion-limited metal electrodeposition. In the intermediate stage, Y_2_O_3_ particles arriving at the cathode surface serve as additional nucleation sites, promoting grain refinement and modifying the growth direction. In the later stage, growth becomes increasingly diffusion-controlled, and the incorporated particles act as barriers to grain boundary migration, further inhibiting grain growth.

**Figure 4:**
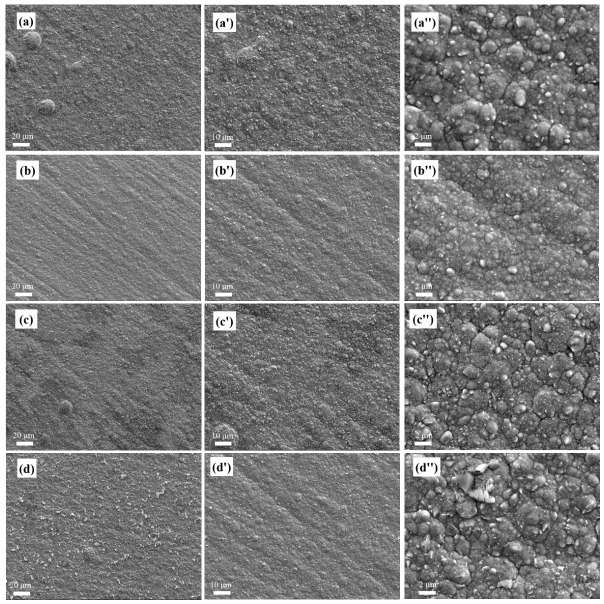
SEM images of the composite coatings, (**a**–**a″**) C0 coating, (**b**–**b″**) C-50 nm coating, (**c**–**c″**) C-1 μm coating, (**d**–**d″**) C-2 μm coating.

Figure 4 presents the SEM images of Ni-W-Y_2_O_3_ composite coatings reinforced with Y_2_O_3_ particles of varying sizes. The composite coatings exhibit a dense structure, free from notable defects such as pores and cracks. As shown in Figure 4a, the Ni-W alloy coating (C0) features a higher number and larger size of cauliflower-like nodules, which is a common characteristic of alloy coatings. The incorporation of Y_2_O_3_ particles into the Ni-W alloy coating modifies this nodule structure, leading to a reduction in both the number and size of the nodules. Consequently, the surface of the composite coatings becomes smoother compared to the C0 coating. Particularly, the C-50 nm composite coating, which includes nanoparticles, demonstrates the best surface morphology and quality, with fewer nodular structures exceeding 5 microns in size, as depicted in Figure 4b. Figure 4b–d confirm the successful incorporation of Y_2_O_3_ reinforcement particles into the composite coatings. Notably, the C-2 μm composite coating, with larger particle sizes, displays the highest number of exposed particles on the surface. Following 5 min of ultrasonic cleaning post-preparation, it is evident that these particles are not loosely adsorbed but rather have not been fully embedded during the deposition process. Enlarged images in Figure 4b″–d″ reveal scattered white needle- and dot-like particles, identified as Y_2_O_3_ particles that remain partially unburied.

**Figure 5:**
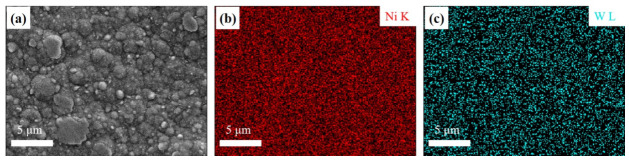
(**a**) SEM morphology of the C0 coating and (**b**,**c**) corresponding EDS elemental mapping of Ni and W.

**Figure 6:**
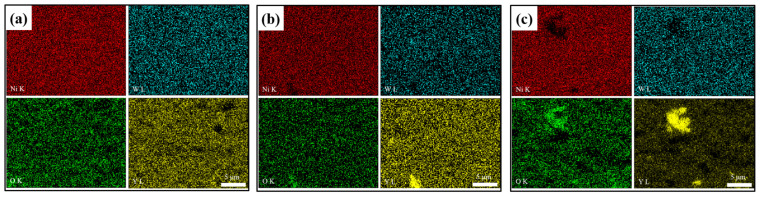
EDS mapping results of different composite coatings, (**a**) C-50 nm, (**b**) C-1 μm, (**c**) C-2 μm.

Figure 5 and Figure 6 present EDS surface scan images of the composite coatings. As depicted in Figure 5, the first picture shows the area where the C0 coating was subjected to EDS analysis. The distribution of Ni and W elements in the Ni-W alloy is notably homogeneous, with no observable regions of element enrichment. In Figure 6a–c, the Y element is uniformly distributed across the entire surface of the composite coating. However, Figure 6b,c reveals localized enrichments of Y, attributable to the larger size of the Y_2_O_3_ particles incorporated. During the composite deposition process, some Y_2_O_3_ particles, which are not fully covered, become exposed on the coating surface. When particles are transported to the cathode surface by convection and electrophoresis, they become trapped by the growing Ni-W deposit. Particles that arrive sufficiently early become fully embedded within the coating matrix, while those that arrive near the end of the deposition process or are too large to be completely covered within the remaining time become partially exposed on the surface. This phenomenon is more pronounced for larger particles, as they require longer deposition times to be fully engulfed, explaining why the C-2 μm coating exhibits the highest density of exposed particles. Overall, the use of nanoscale Y_2_O_3_ particles offers significant benefits in terms of achieving a more uniform distribution.

Incorporating different Y_2_O_3_ particle sizes into the electrodeposition solution affects the deposition rate of the constituent elements in the composite coating. According to the data presented in Table 2, the C0 coating contains 73.68% Ni and 26.32% W. The introduction of Y_2_O_3_ particles results in an increase in the W mass fraction and a corresponding decrease in the Ni content. This effect is attributed to the constant current DC electrodeposition technique used, where Y_2_O_3_ particle adsorption on the cathode surface reduces the active cathode area, thereby shifting the reduction potential of Ni-W to more negative values. A more negative reduction potential favors W deposition [38]. Notably, in the C-2 μm composite coating with 2 μm Y_2_O_3_ particles, the change in element composition is pronounced: Ni content decreases to 62.08%, while W content increases to 30.23%. The total mass fraction of metal elements in the C-2 μm coating is lower compared to other coatings, while the comprehensive mass fraction of Y_2_O_3_ particles is higher. This discrepancy is due to the larger 2 μm particle size, which increases the likelihood of incomplete coating, resulting in a higher observed mass fraction of Y_2_O_3_ in the EDS analysis.

It should be noted that the EDS maps presented in Figure 6 are qualitative representations of elemental distribution and are intended to illustrate the uniformity of Y distribution, rather than to enable quantitative comparison between different coatings. The apparent differences in signal intensity between maps arise from variations in the selected scan areas, local particle densities, and detection geometry. Quantitative Y content is correctly provided by the EDS compositional analysis in Table 2, which shows that the C-2 μm coating contains 5.45 wt.% Y, significantly higher than the C-1 μm coating (1.93 wt.%). The prominent Y signals observed in the maps reflect the successful incorporation and distribution of Y_2_O_3_ particles, consistent with the quantification results.

### 3.3. Crystalline Phase Analysis

**Figure 7:**
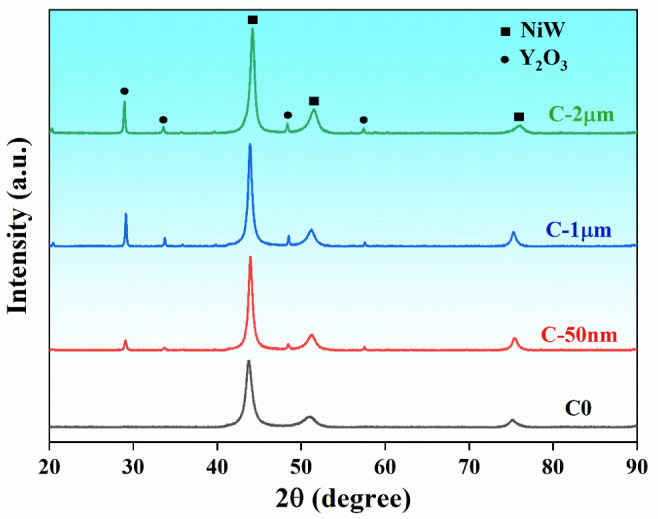
XRD patterns of the Ni-W-Y_2_O_3_ composite coatings.

Figure 7 presents the XRD patterns of the composite coatings. The diffraction peaks were identified using ICDD PDF No. 00-004-0850 for the Ni-W matrix and ICDD PDF No. 00-041-1105 for Y_2_O_3_ particles. The patterns reveal that the coatings are composed of a single-phase Ni-W alloy matrix with a face-centered cubic (FCC) structure. The diffraction peaks at 44.3°, 51.7°, and 76.1°, denoted by black squares, correspond to the Ni-W (111), (200), and (220) planes, respectively. Compared to the standard diffraction peaks for pure Ni at 44.5°, 51.8°, and 76.3°, the observed peaks are slightly shifted to lower angles. This shift is attributed to the formation of a tungsten solid solution (α-Ni-W) within the FCC Ni matrix, leading to lattice expansion due to tungsten dissolution. It should be noted that while the W content varies among the four coatings, the variation remains within the solid solubility limit of W in Ni, which is approximately 37 at%. Consequently, no additional phases are expected to form. The absence of significant changes in peak positions and intensities can be attributed to the relatively small composition variation, the nanocrystalline grain size, and the influence of microstrain and texture on peak broadening. These factors collectively mask any subtle lattice parameter changes that might arise from composition variation. Additionally, the XRD patterns exhibit diffraction peaks of Y_2_O_3_ particles, marked by black dots, confirming their successful incorporation into the composite coating. Importantly, the addition of Y_2_O_3_ particles does not alter the single-phase FCC structure of the Ni-W matrix. The intensity of the Y_2_O_3_ peaks, particularly the (222) plane at 2θ = 29.2°, increases with larger Y_2_O_3_ particle sizes. This trend aligns with observations from the surface topography in Figure 4, where larger particles are more likely to remain partially exposed, contributing to increased peak intensity in the XRD analysis. The intensity of the Ni-W (111), (200), and (220) peaks remains unchanged, indicating that Y_2_O_3_ incorporation does not affect the FCC structure of the Ni-W alloy matrix.

**Figure 8:**
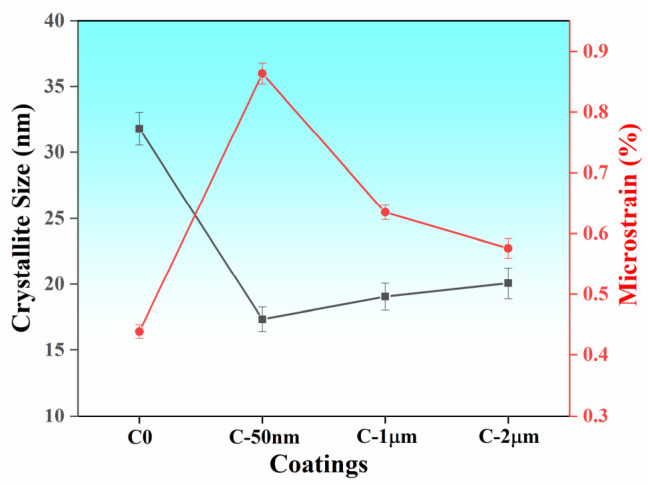
Crystallite size and micro-strain of the composite coatings.

Figure 8 illustrates the influence of Y_2_O_3_ particle size on the crystallite size and microstrain of Ni-W-Y_2_O_3_ composite coatings. The (111) diffraction peak of these composite coatings was isolated, and the crystallite size and microstrain were determined using the Voigt single peak method. According to Formula 2, crystallite size (D) is inversely proportional to β(111), while microstrain (ε) is directly proportional to β(111). Therefore, the linear broadening of the diffraction peak is closely associated with both the crystallite size and the microstrain. The results indicate that all composite coatings are composed of nano-sized grains and exhibit relatively high microstrain. Specifically, the crystallite sizes for C0, C-50 nm, C-1 μm, and C-2 μm coatings are 31.8 nm, 17.3 nm, 19.0 nm, and 20.1 nm, respectively. The incorporation of micro/nano-sized Y_2_O_3_ particles refines the crystallite size of the composite coatings, with nano-sized Y_2_O_3_ particles achieving a more pronounced refinement compared to micro-sized particles. Conversely, microstrain exhibits an opposite trend to crystallite size; the microstrain in coatings containing Y_2_O_3_ particles is elevated relative to the C0 coating, with smaller crystallite sizes correlating to greater microstrain in the composite coatings.

**Figure 9:**
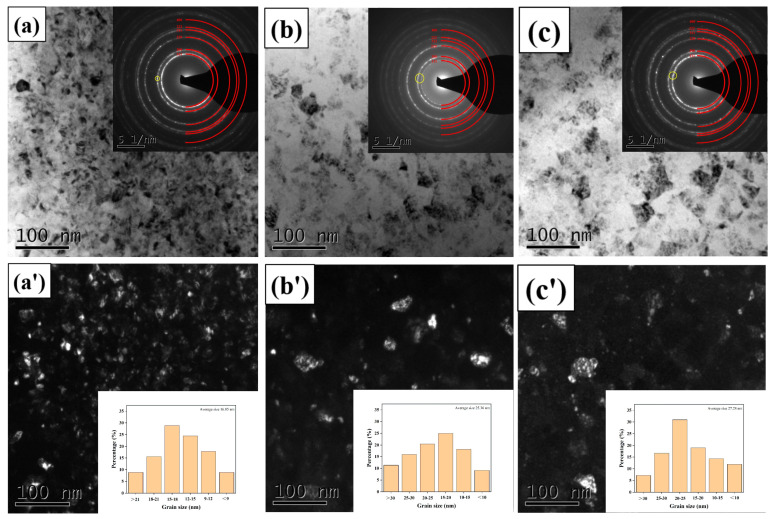
TEM images of different composite coatings under bright and dark field and the selected area diffraction pattern, (**a**,**a′**) C-50 nm coating, (**b**,**b′**) C-m coating, (**c**,**c′**) C-2 μm coating.

Figure 9 illustrates the TEM bright-field and dark-field images, along with selected electron diffraction patterns of Ni-W-Y_2_O_3_ composite coatings. Analysis reveals that the interior of the composite coating is nanocrystalline, characterized by a fine, dispersed, and uniformly distributed microstructure. The selected electron diffraction patterns confirm that the coatings possess a single-phase FCC structure, consistent with the XRD results. In the bright-field image, dark spots correspond to grains with varying growth orientations. Notably, there is a direct correspondence between the grains observed in the bright-field image and those in the dark-field image. The grain size data is presented in a histogram, which facilitates a rapid assessment of particle size and frequency distribution at the nanoscale. The histogram indicates that the grain size distribution of the composite coatings adheres approximately to a normal distribution. Specifically, the average grain sizes for C-50 nm, C-1 μm, and C-2 μm coatings are 16.85 nm, 25.36 nm, and 27.28 nm, respectively. These values are in alignment with the crystallite sizes determined from the XRD patterns. Discrepancies between XRD and TEM measurements are attributed to the differing scales of grain statistics: TEM provides direct observation but involves a smaller number of grains due to its limited field of view, while XRD covers a larger scanning range, thus encompassing more grains and offering a more comprehensive statistical representation. Comparative analysis reveals that the composite coating reinforced with nano-sized Y_2_O_3_ particles exhibits the smallest grain size and the most uniform microstructure. In contrast, coatings reinforced with micro-sized Y_2_O_3_ particles show greater grain size variation and less uniform microstructural properties.

### 3.4. Electrochemical Corrosion Properties

The incorporation of micro/nano-sized Y_2_O_3_ particles notably influences the microstructural evolution and corrosion resistance of Ni-W-Y_2_O_3_ composite coatings. The presence of these particles not only refines the microstructure of the Ni-W-Y_2_O_3_ coatings, resulting in a smoother surface, but also affects the corrosion behavior of the Ni-W coatings, thereby altering the overall corrosion resistance of the composite coatings. He et al. [39] elucidate that the enhancement in corrosion performance due to particle addition is twofold: Firstly, the incorporation of nano-sized Y_2_O_3_ particles modifies the surface chemistry and energy state of the composite coatings. Secondly, the homogeneous dispersion of particles strengthens the coating matrix, prevents its dissolution, and substantially improves the coating’s corrosion resistance. Additionally, the size and concentration of the reinforcing particles play a critical role in determining the corrosion resistance of the composite coatings [40].

#### 3.4.1. Corrosion Properties in Acidic Environment

**Figure 10:**
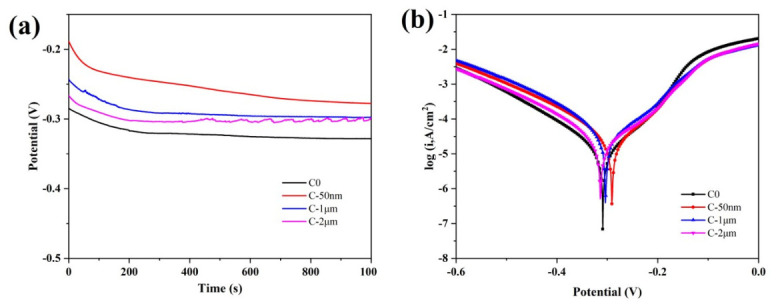
(**a**) Open-circuit potential curves vs. time and (**b**) Tafel curves of the Ni-W-Y_2_O_3_ composite coatings in 10 wt.% H_2_SO_4_ solution.

Figure 10 illustrates the open-circuit potential and Tafel polarization curves of Ni-W-Y_2_O_3_ composite coatings in a 10 wt.% H_2_SO_4_ acidic solution. It is evident from the figure that the C0 coating, which lacks Y_2_O_3_ particles, exhibits the lowest open-circuit potential. The incorporation of micro/nano Y_2_O_3_ particles enhances the open-circuit potential, with nano-sized Y_2_O_3_ particles imparting the highest potential. The Tafel polarization curves, shown in Figure 10b, reveal that in the acidic environment, the self-corrosion potential of the C-50 nm and C-1 μm coatings is higher than that of the C0 and C-2 μm coatings, indicating superior corrosion performance for the former two. It should be noted that the effect of Y_2_O_3_ particle size on corrosion resistance does not follow a simple monotonic trend. Instead, a clear threshold behavior is observed: nano-sized particles provide significant improvement, while micron-sized particles exhibit marginal or even negative effects depending on the corrosive environment. This size-dependent behavior is governed by the balance between defect filling/refinement and the introduction of interfacial defects, which favor different size regimes. Table 3 provides the corrosion potential (E_corr_) and corrosion current density (I_corr_) of these composite coatings as determined by Tafel extrapolation. The C0 coating exhibits an E_corr_ of −315 mV and an I_corr_ of 4.85 μA/cm^2^. The corrosion potential increases and the corrosion current density decreases with the addition of nano-sized Y_2_O_3_ particles, thus enhancing the corrosion resistance of the composite coating in acidic conditions. In contrast, the introduction of micron-sized Y_2_O_3_ particles results in minimal changes in corrosion potential and current density, showing negligible improvement compared to the C0 coating. The C-2 μm coating exhibits the highest corrosion current density in acidic solution, indicating the poorest corrosion resistance among the composite coatings. This is attributed to the partial exposure and incomplete embedding of the larger Y_2_O_3_ particles at the coating surface. The exposed particles generate interfacial gaps and defects that serve as preferential sites for corrosive attack. Additionally, the larger particles create surface heterogeneity, promoting localized galvanic coupling between Y_2_O_3_-enriched regions and the particle-depleted Ni-W matrix. These mechanisms accelerate anodic dissolution in the acidic environment. In the acidic H_2_SO_4_ solution, the C0 and C-2 μm coatings exhibit comparable corrosion resistance. This equivalence arises because the aggressive acidic environment promotes active dissolution of the Ni-W matrix, overwhelming any potential beneficial effects of the Y_2_O_3_ particles. For the C-2 μm coating, the larger particles introduce interfacial gaps and micro-defects that facilitate electrolyte penetration, counteracting any beneficial effect. For the C0 coating, the absence of particles means no such defects are introduced. The net result is comparable corrosion performance for both coatings under these aggressive conditions.

**Figure 11:**
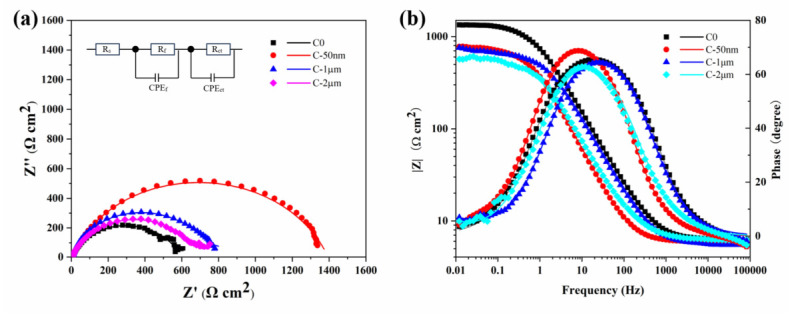
(**a**) Nyquist plots, the equivalent circuit mode, and (**b**) Bode plots of the Ni-W-Y_2_O_3_ composite coatings in 10 wt.% H_2_SO_4_ solution.

ZSimpWin software V 3.30 was employed to analyze the data from electrochemical impedance spectroscopy (EIS) tests, resulting in Nyquist and Bode plots, as illustrated in Figure 11. The dotted lines represent experimental data, while the solid lines denote the fitted curves. The shape and radius of the Nyquist plot arcs are indicative of the corrosion performance of the coating. A single arc suggests that the corrosion mechanism is primarily governed by charge transfer, with a larger arc radius reflecting enhanced corrosion resistance. As shown in Figure 11a, all coatings exhibit a single arc. In the Nyquist plot, the arc radius is a direct reflection of the charge transfer resistance. A larger arc radius corresponds to a higher charge transfer resistance, indicating that the electrochemical reaction at the electrode/electrolyte interface is more kinetically hindered. This kinetic hindrance implies a slower corrosion rate and superior corrosion protection. The relationship is grounded in the principle that charge transfer resistance is inversely proportional to the corrosion current density, providing a direct correlation between the arc radius and the corrosion performance. The incorporation of Y_2_O_3_ particles increases the arc radius, demonstrating improved corrosion resistance of the composite coating in acidic solutions, although it does not alter the underlying corrosion mechanism. The C0 coating displays the smallest arc radius, signifying the poorest corrosion resistance. The addition of micron-sized Y_2_O_3_ particles improves corrosion resistance to some extent, but the effect is more pronounced with nano-sized Y_2_O_3_ particles, which significantly enhance the arc radius of the Nyquist plot, indicating superior corrosion resistance of the C-50 nm coating. The Bode plots in Figure 11b provide complementary information to the Nyquist analysis. In the high-frequency region above 10^3^ Hz, the impedance magnitudes of all coatings are similar, reflecting the solution resistance and the rapid dielectric response of the coating surface. In the mid-frequency region, the impedance magnitude becomes dependent on the coating properties, with the C-50 nm coating showing the highest impedance, indicating superior barrier performance. In the low-frequency region below 1 Hz, the impedance magnitude approaches the polarization resistance. The C-50 nm coating exhibits the highest low-frequency impedance, confirming its best corrosion resistance. The phase angle plots further show that the C-50 nm coating maintains a phase angle closer to −90° over a broader frequency range, indicating more ideal capacitive behavior and a more stable passive film.

The illustration in Figure 11a shows the equivalent circuit diagram used in Nyquist curve fitting, where R_s_ is the solution resistance of the corrosive medium, CPE_f_ and CPE_ct_ are the non-ideal capacitance between the solid-liquid interface of the coating and the corrosive medium and the solid-solid interface between the coating and the substrate, respectively. R_f_ and R_ct_ are charge transfer resistors at the solid-liquid interface and the solid-solid interface, respectively [41,42]. As detailed in Table 4, the unmodified C0 coating exhibits the lowest charge transfer resistances, with R_f_ and R_ct_ measured at 490.3 Ω cm^2^ and 189.2 Ω cm^2^, respectively. Upon the incorporation of Y_2_O_3_ particles, an increase in R_f_ and R_ct_ is observed in the composite coatings. Notably, the addition of nano-sized Y_2_O_3_ particles results in a significant enhancement of resistance. Specifically, the C-50 nm coating shows markedly increased R_f_ and R_ct_ values of 732.2 Ω cm^2^ and 1342.0 Ω cm^2^, respectively. This is because the nanoparticles effectively fill pores and defects, reducing the penetration pathways for the corrosive electrolyte. The R_ct_ values increase after incorporating nano particles, indicating that charge transfer at the electrode-electrolyte interface is significantly impeded, which is attributed to the grain refinement effect and the formation of a more stable passive film. For the C-1 μm coating, the R_f_ and R_ct_ values are intermediate, reflecting the partial beneficial effect of the micron-sized particles. For the C-2 μm coating, the values are close to those of the C0 coating, confirming the limited improvement due to the introduction of interfacial defects. The CPE values, particularly the higher CPE_ct_ for the C-2 μm coating, indicate increased surface heterogeneity, consistent with the presence of exposed particles and interfacial gaps. This demonstrates that nano-sized Y_2_O_3_ particles more effectively improve the corrosion resistance of Ni-W-Y_2_O_3_ composite coatings in acidic corrosive environments compared to micron-sized particles.

#### 3.4.2. Corrosion Properties in Neutral Environment

**Figure 12:**
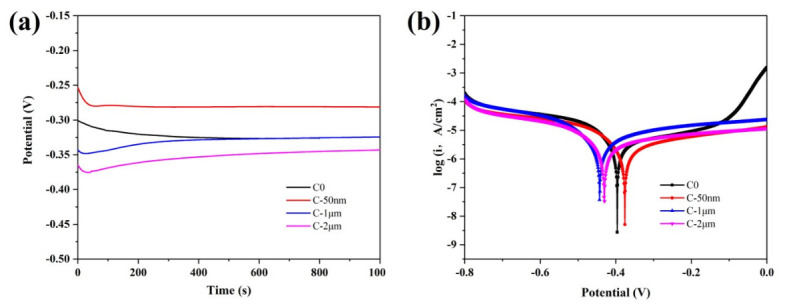
(**a**) Open-circuit potential curves vs. time and (**b**) Tafel curves of the composite coatings in 3.5 wt.% NaCl neutral solution.

In Figure 12, the open-circuit potential and Tafel polarization curves of Ni-W-Y_2_O_3_ composite coatings in 3.5 wt.% NaCl solution are presented. The addition of Y_2_O_3_ particles of varying sizes affects the open-circuit potential differently. Specifically, the open-circuit potential of the C-50 nm coating shifts positively, whereas the C-1 μm and C-2 μm coatings exhibit a negative shift compared to the C0 coating. Table 5 summarizes the corrosion potential (E_corr_) and corrosion current density (I_corr_) for each coating derived from Tafel extrapolation. For the C0 coating, E_corr_ is −398 mV, and I_corr_ is 4.35 μA/cm^2^. The C-50 nm coating shows an improved corrosion performance with E_corr_ increasing to −382 mV and I_corr_ decreasing to 3.53 μA/cm^2^. Conversely, the addition of micron-sized Y_2_O_3_ particles results in a decrease in corrosion potential and an increase in corrosion current density, indicating reduced corrosion resistance. This finding contradicts the literature [43], which reports that micron-sized particles enhance corrosion resistance by refining the grain size and eliminating the (200) texture in pure Ni coatings. In contrast, the Ni-W alloy matrix in this study did not develop a strong texture during growth. Consequently, while micrometer-sized Y_2_O_3_ particles refine the crystallite size, their larger size introduces increased surface composition fluctuations, which exacerbate corrosion susceptibility in these areas.

**Figure 13:**
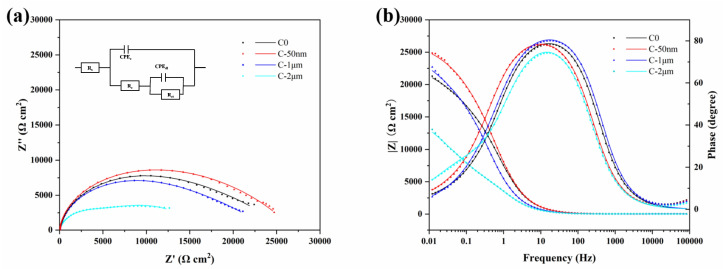
(**a**) Nyquist plots, the equivalent circuit mode, and (**b**) Bode plots of the Ni-W-Y_2_O_3_ composite coatings in 3.5 wt.% NaCl neutral solution.

The Nyquist and Bode plots of Ni-W-Y_2_O_3_ composite coatings reinforced with particles of different sizes in a neutral solution of 3.5 wt.% NaCl is shown in Figure 13. The Nyquist curve of C0 coating, C-50 nm coating, and C-1 μm coating is a single approximate semi-circular arc, and the arc radius of C-50 nm coating is the largest, indicating that its corrosion resistance is the best; the arc radius of C-1 μm coating is smaller than that of C0 coating, indicating that its corrosion resistance is decreased. The Nyquist curve of the 2 μm Y_2_O_3_ particle reinforced C-2 μm coating is obviously different from that of other coatings, and the corrosion reaction process is more complicated. It can be seen from the Bode plots in Figure 13b that the charge transfer resistance of each composite coating is basically the same in the middle and high frequency region; however, in the low frequency region, the charge transfer resistance of the C-2 μm coating suddenly drops sharply, and the corrosion process occurs rapidly, resulting in a serious decline in its corrosion resistance. This behavior is attributed to the presence of interfacial gaps and micro-defects introduced by the partially embedded 2 μm Y_2_O_3_ particles, which serve as preferential pathways for electrolyte penetration and accelerate anodic dissolution. The C-50 nm and C-1 μm coatings exhibit higher low-frequency impedance, suggesting more effective barrier properties and slower charge transfer kinetics. The phase value of C-2 μm in the Bode-phase figure is also lower than that of other coatings, which also indicates that the corrosion resistance of the coating is relatively poor.

The EIS equivalent circuit diagram of Ni-W-Y_2_O_3_ composite coating in a neutral solution of 3.5 wt.% NaCl is illustrated in Figure 13a. The corrosion parameters derived from fitting the Nyquist plot are summarized in Table 6. The results confirm that, compared to the C0 coating, the charge transfer resistance (R_ct_) and coating resistance (R_c_) of the C-50 nm composite coating increased from 1.23 × 10^4^ Ω cm^2^ and 1.19 × 10^4^ Ω cm^2^ to 1.38 × 10^4^ Ω cm^2^ and 1.36 × 10^4^ Ω cm^2^, respectively. Conversely, the R_ct_ values for both the C-1 μm and C-2 μm coatings decreased. Notably, the R_c_ for the C-2 μm coating fell to 0.53 × 10^4^ Ω cm^2^, indicating a higher susceptibility to corrosion and a decline in corrosion resistance. These findings are consistent with the results from the Tafel polarization curve extrapolation method. The addition of Y_2_O_3_ particles of different sizes has a significant impact on the corrosion performance of Ni-W-Y_2_O_3_ composite coatings in neutral saline solutions. Specifically, the incorporation of nano-sized Y_2_O_3_ particles enhances the corrosion resistance of the composite coating, whereas the use of micron-sized Y_2_O_3_ particles leads to a deterioration in corrosion resistance.

The particle size of Y_2_O_3_ significantly influences the corrosion performance of Ni-W-Y_2_O_3_ composite coatings. In both acidic and neutral corrosive environments, micrometer-sized Y_2_O_3_ particles fail to enhance the corrosion resistance of these coatings effectively. In contrast, nano-sized Y_2_O_3_ particles markedly improve the corrosion performance of the composite coatings. This enhancement is attributed to the nanoparticles’ ability to fill nanoscale cracks within the coating matrix [44,45]. The presence of nano-sized particles mitigates coating defects, refines the microstructure, and thus enhances overall corrosion resistance [22,23,46]. Incorporating nanoparticles not only promotes grain refinement but also addresses surface irregularities such as gaps and grooves in the Ni-W matrix, functioning as a physical barrier that inhibits the progression of corrosion. To be more specific, the particle-size-dependent corrosion behavior of Ni-W-Y_2_O_3_ composite coatings can be understood from three interconnected aspects. First, from the perspective of defect density, nano-sized Y_2_O_3_ particles can effectively fill the micro-pores, inter-granular gaps, and surface grooves inherently present in the electrodeposited Ni-W matrix. By occupying these defect sites, the nanoparticles reduce the effective area of direct electrolyte-coating contact, thereby suppressing localized dissolution and pitting initiation. In contrast, micron-sized particles, due to their larger dimensions, cannot enter nano-scale defects; instead, they tend to accumulate at grain boundaries or remain partially embedded, creating new interfacial gaps that act as preferential channels for corrosive species infiltration. Second, regarding the passive film stability, the incorporation of Y_2_O_3_ particles modifies the surface chemistry of the composite coating. Nano-sized Y_2_O_3_, with its high specific surface area and uniform dispersion, promotes the formation of a more compact and continuous WO_3_- and Ni(OH)_2_-enriched passive film by providing numerous nucleation sites for passive layer growth. The refined grain structure from 31.8 nm to 17.3 nm also contributes to higher grain boundary density, which facilitates rapid chromium-free passive film formation and repair. In contrast, micron-sized particles cannot provide such a uniform dispersion effect; they create compositional heterogeneity on the surface, leading to local galvanic coupling between the Y_2_O_3_-enriched regions and the particle-depleted Ni-W matrix, which accelerates anodic dissolution in neutral chloride media. Third, regarding the electrochemical response in different media, in acidic H_2_SO_4_ solution, the dominant degradation mechanism is hydrogen evolution and active dissolution of the Ni-W matrix. The physical barrier effect of particles is less significant because the acidic environment aggressively attacks the matrix regardless of particle size. However, nano-sized Y_2_O_3_ still provides moderate protection by reducing the real active surface area through defect filling, resulting in a slightly higher charge transfer resistance. In a neutral NaCl solution, the corrosion mechanism shifts to chloride-induced pitting. Here, the passive film stability becomes the dominant factor. Nano-sized Y_2_O_3_ enhances the passive film resistance to chloride attack, while micron-sized particles disrupt passive film continuity and create pit initiation sites, explaining why C-1 μm and C-2 μm coatings exhibit degraded corrosion resistance in neutral media. R_c_ dropped to 1.15 × 10^4^ and 0.53 × 10^4^ Ω cm^2^, respectively, compared to 1.38 × 10^4^ Ω cm^2^ for C-50 nm. This size-dependent mechanism explains why nano-sized Y_2_O_3_ is universally beneficial, whereas micron-sized Y_2_O_3_ is only neutral or even detrimental depending on the corrosive environment. Consequently, the use of nano Y_2_O_3_ particles significantly enhances the corrosion resistance of Ni-W-Y_2_O_3_ composite coatings. Similarly, research by Wang et al. [28] demonstrated that SiO_2_ nanoparticles improve the corrosion resistance of Ni-W coatings by acting as physical barriers that fill surface pores and voids.

### 3.5. Microhardness

**Figure 14:**
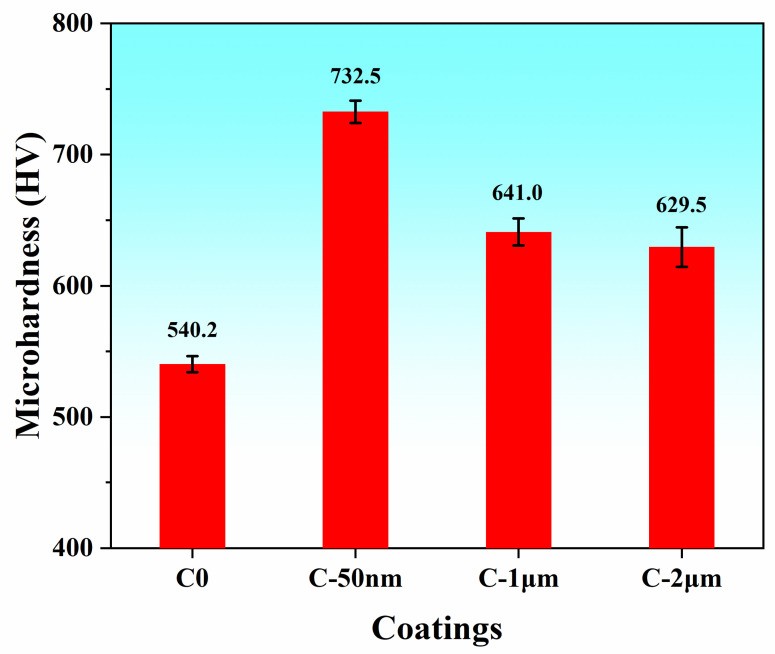
Surface microhardness values of the Ni-W-Y_2_O_3_ coatings.

Figure 14 depicts the microhardness value of the Ni-W-Y_2_O_3_ composite coating surfaces. The base coating (C0) exhibits a microhardness of 540.2 HV. The microhardness significantly increases with the addition of Y_2_O_3_ particles, with the C-50 nm coating demonstrating the highest value of 732.5 HV. The microhardness of C-1 μm and C-2 μm coatings reached 641.0 HV and 629.5 HV, respectively. The incorporation of micro/nano Y_2_O_3_ particles improves the hardness from two aspects: the dispersion strengthening effect of the Y_2_O_3_ particles and the refinement of the coating’s microstructure. The incorporation of Y_2_O_3_ particles not only strengthens the composite coating through dispersion but also promotes grain refinement and optimizes the nodular structure on the composite coating surface, resulting in a denser and more robust surface morphology, thus significantly increasing the microhardness of the particle-reinforced Ni-W-Y_2_O_3_ composite coatings. The microhardness values presented in Figure 14 are mean values with standard deviations. The differences among the four coatings are consistent and reproducible across multiple samples.

Reinforcing particle size significantly influences the effectiveness of dispersion strengthening. Compared to micron-sized Y_2_O_3_ particles, nano-sized Y_2_O_3_ particles provide a superior dispersion strengthening effect. This study focuses on the fine-grain strengthening mechanism. According to the Hall-Petch relationship, a reduction in grain size generally leads to increased hardness. Incorporating micro/nano Y_2_O_3_ particles into Ni-W-Y_2_O_3_ composite coatings results in a notable decrease in crystallite size, with the coatings exhibiting nano-scaled crystallites. It has been reported [47,48] that when crystallite sizes range between 10 and 35 nm, the microhardness of the Ni-W alloy coating aligns with the Hall-Petch formula. However, when crystallite sizes fall below 10 nm, the coating tends to soften, and hardness decreases with further decreases in crystallite size. Thus, while refining grain size can enhance hardness, excessive refinement may be counterproductive. Typically, higher hardness is associated with better wear resistance, and enhancing material hardness is a common approach to improving wear resistance.

### 3.6. Wear Behaviors

**Figure 15:**
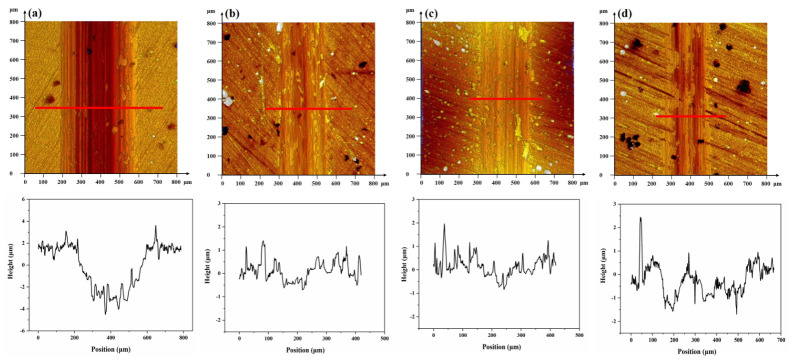
Three-dimensional topographies and cross-sectional depth profiles of wear traces of the composite coatings, (**a**) C0 coating, (**b**) C-50 nm coating, (**c**) C-1 μm coating, (**d**) C-2 μm coating.

The wear resistance was evaluated based on the width and depth of the wear marks. Figure 15 presents the three-dimensional morphology and cross-sectional profiles of wear marks on Ni-W-Y_2_O_3_ composite coatings after wear resistance testing. Observations reveal pronounced plow grooves, scratches, and adhesive tearing across all coatings. Quantitative analysis of the wear track cross-sectional profiles reveals that the C0 coating exhibits the widest and deepest wear track, with a width of approximately 485 μm and a depth of about 8.2 μm. In contrast, the C-50 nm coating shows the narrowest and shallowest wear track, measuring roughly 320 μm in width and 5.1 μm in depth, which corresponds to its highest hardness and lowest wear rate. The C-1 μm and C-2 μm coatings display intermediate values, with widths and depths of approximately 335 μm/5.3 μm and 380 μm/6.4 μm, respectively, consistent with their intermediate wear performance. Notably, the wear cross-sectional profiles of the C-50 nm and C-1 μm coatings are strikingly similar, with significantly reduced width and depth of wear marks compared to other samples. The C-2 μm coating shows a moderate level of wear resistance. These findings suggest that Y_2_O_3_ particles of 1 μm and 50 nm in size confer superior wear resistance to the composite coatings.

**Figure 16:**
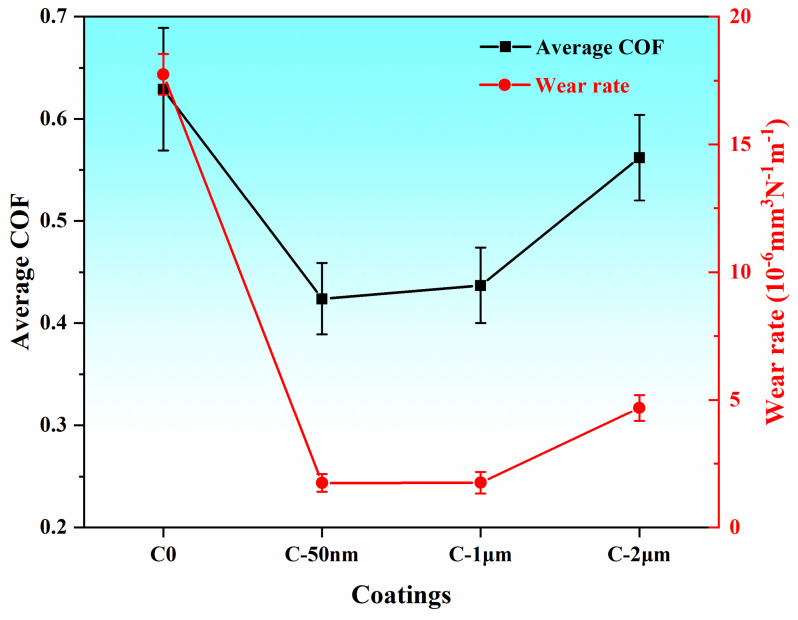
Average friction coefficient and wear rate of the composite coatings.

In Figure 16, the average friction coefficient and wear rate are illustrated, with the wear rate calculated from the cross-sectional profile of the wear tracks, their length, wear distance, and applied load. The data reveal that the C0 coating exhibits the highest average friction coefficient (0.63) and wear rate (17.75 × 10^−6^ mm^3^ N^−1^ m^−1^). In contrast, the C-50 nm and C-1 μm coatings show lower and nearly identical average friction coefficients and wear rates. The C-2 μm coating also demonstrates an increased friction coefficient and wear rate compared to the C0 coating. This suggests that incorporating micro/nano Y_2_O_3_ particles improves the wear resistance of the composite coatings to varying extents. This enhancement is attributed to the increased hardness and denser surface morphology resulting from the addition of Y_2_O_3_ particles, which refines the grain structure and shifts the wear mechanism from predominantly adhesive wear in the C0 coating to a combination of adhesive and abrasive wear in the composite coatings. Consequently, Ni-W-Y_2_O_3_ composite coatings exhibit superior wear resistance compared to the pure Ni-W alloy coating, and the average friction coefficient and wear rate of the C-2 μm coating are slightly increased. However, it is still better than the performance of the C0 coating. It shows that the incorporation of micro/nano Y_2_O_3_ particles can enhance the wear resistance of the composite coatings to different degrees.

The wear resistance of composite coatings is closely related to their hardness and microstructure. The effect of Y_2_O_3_ particle size on the wear resistance of Ni-W-Y_2_O_3_ composite coatings can be rationalized through three interrelated mechanisms: particle-induced hardening, particle embedding and pull-out behavior, and debris-mediated friction modulation. The first mechanism concerns the hardness enhancement conferred by Y_2_O_3_ particles of different sizes. The composite coatings containing nano-sized Y_2_O_3_ particles exhibit the highest microhardness of 732.5 HV, while the C-1 μm and C-2 μm coatings show hardness values of 641.0 HV and 629.5 HV, respectively, all considerably higher than the 540.2 HV measured for the particle-free C0 coating. The hardness improvement originates from two synergistic contributions. On the one hand, the finely dispersed Y_2_O_3_ particles act as effective obstacles to dislocation motion, providing Orowan strengthening. On the other hand, the incorporation of Y_2_O_3_ particles promotes grain refinement of the Ni-W matrix, as evidenced by the reduction in crystallite size from 31.8 nm in the C0 coating to 17.3 nm in the C-50 nm coating, which results in Hall-Petch strengthening. The smaller the reinforcing particles, the more pronounced the grain refinement effect and thus the higher the hardness. This increase in surface hardness directly translates into enhanced resistance to plastic deformation and groove formation during sliding contact, which is the primary reason why the C-50 nm coating exhibits the best wear resistance among all coatings.

The second mechanism involves the reinforcing behavior of Y_2_O_3_ particles during the wear process, which is strongly dependent on particle size and the degree of particle embedding within the Ni-W matrix. Nano-sized Y_2_O_3_ particles are generally well dispersed and firmly embedded in the coating matrix due to their high specific surface area and uniform distribution. During sliding, these firmly embedded nanoparticles act as effective load-bearing elements that resist plowing and cutting by the counterbody, thereby reducing wear volume. In contrast, micron-sized particles, especially the 2 μm particles, exhibit a greater tendency for partial exposure at the coating surface. This is because larger particles are more difficult to be fully engulfed by the growing Ni-W deposit during electrodeposition, as confirmed by the SEM observations in Figure 4 showing numerous exposed particles on the C-2 μm coating surface. Under the normal load applied during wear testing, these partially exposed particles experience high stress concentration at the particle-matrix interface. The stress concentration eventually leads to microcrack initiation at the interface and subsequent particle pull-out. Once dislodged, these hard particles become trapped between the sliding surfaces. The vacant sites left behind by the pulled-out particles increase surface roughness and promote more severe material removal in subsequent sliding passes.

The third mechanism relates to the role of wear debris in modifying the friction and wear behavior. During sliding, Y_2_O_3_ particles that become partially dislodged but remain at the contact interface can participate in rolling friction. This rolling action effectively reduces the shear stress at the sliding interface and lowers the friction coefficient. Indeed, the average friction coefficient decreases from 0.63 for the C0 coating to 0.52 for both the C-50 nm and C-1 μm coatings. The reduced friction coefficient, combined with the higher hardness, leads to significantly lower wear rates for these composite coatings compared to the pure Ni-W alloy coating. However, for the C-2 μm coating, the larger dislodged particles cause more severe scratching and micro-cutting on the worn surface, resulting in a slightly higher friction coefficient of 0.59 and a correspondingly higher wear rate than the C-50 nm and C-1 μm coatings. Nevertheless, the wear rate of the C-2 μm coating remains lower than that of the C0 coating, indicating that even micron-sized Y_2_O_3_ particles provide some beneficial effect, albeit less pronounced than that of the smaller particles.

Based on the above analysis, the wear mechanism transition from adhesive wear to mixed adhesive-abrasive wear can be understood as a function of particle size. In the particle-free C0 coating, the wear surface exhibits extensive plastic deformation and laminar spalling characteristic of adhesive wear, which is attributed to the high metallic affinity of the nickel-tungsten alloy and the accumulation of transferred material on the counterbody surface. After the incorporation of Y_2_O_3_ particles, the wear mechanism shifts to a combination of adhesive and abrasive wear. For nano-sized particles, the abrasive component is mild and accompanied by beneficial rolling friction, leading to the highest wear resistance. For micron-sized particles, the abrasive component becomes more severe due to larger debris size and more pronounced particle pull-out, but the overall wear resistance remains superior to that of the unreinforced coating. This particle size-dependent mechanistic framework provides a comprehensive explanation for the observed wear behavior and offers practical guidance for selecting the appropriate Y_2_O_3_ particle size in Ni-W composite coating design.

**Figure 17:**
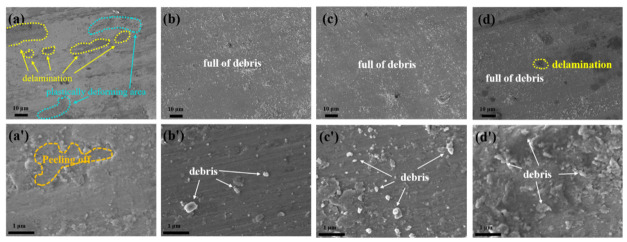
SEM morphologies of the worn surface, (**a**,**a′**) C0 coating, (**b**,**b′**) C-50 nm coating, (**c**,**c′**) C-1 μm coating, (**d**,**d′**) C-2 μm coating.

Figure 17 presents SEM images of wear tracks obtained after conducting wear resistance testing. The SEM images reveal the presence of furrows, scratches, and adhesive tear marks on the wear tracks. In Figure 17a,a′, the wear surface of the C0 coating exhibits significant plastic deformation along with extensive laminar spalling and adhesive wear marks. This phenomenon is attributed to the high viscosity of Ni metal and the accumulation of spalled material on the surface of the steel ball, resulting in a dominant adhesive wear mechanism for the C0 coating. In contrast, Figure 17b–d,b′–d′ depict the wear surfaces of the C-50 nm, C-1 μm, and C-2 μm coatings, which are covered by a layer of wear debris. This debris is a result of the detachment of Y_2_O_3_ particles from the Ni-W matrix and the subsequent brittle fracture of the matrix material during wear testing. The wear mechanism for these particle-reinforced Ni-W-Y_2_O_3_ composite coatings is characterized by both adhesive and abrasive wear. The incorporation of micro/nano-sized Y_2_O_3_ particles enhances the cross-linking density and hardness of the Ni-W-Y_2_O_3_ composite coatings, thereby improving their wear resistance [36]. Detailed examination of the wear tracks reveals that the C-50 nm and C-1 μm coatings exhibit more debris and fewer laminar spalling and adhesive marks compared to the C-2 μm coating. This observation can be attributed to the denser surface structure and reduced surface nodule formation of the former two coatings, which minimizes large-scale peeling and adhesive wear. Additionally, the smaller size of the Y_2_O_3_ particles in these coatings facilitates their detachment from the matrix, enabling them to participate more effectively in rolling friction and thereby further enhancing wear resistance.

The transition from adhesive wear to mixed adhesive-abrasive wear in Ni-W-Y_2_O_3_ composite coatings can be understood from three interconnected perspectives. Firstly, the particle-free C0 coating exhibits extensive plastic deformation and laminar spalling, with wear debris consisting primarily of metallic flakes originating from the Ni-W matrix. The high metallic affinity of nickel promotes material transfer to the counterbody surface, which is characteristic of adhesive wear. After the incorporation of Y_2_O_3_ particles, the wear debris probably becomes a mixture of metallic fragments and hard ceramic particles. For the C-50 nm coating, the debris particles are fine and evenly distributed, which facilitates rolling friction at the sliding interface and reduces the effective shear stress. For the C-2 μm coating, the larger detached particles generate more severe scratching and micro-cutting, contributing to a more pronounced abrasive component.

Secondly, the interfaces between Y_2_O_3_ particles and the Ni-W matrix serve as preferential sites for microcrack initiation under cyclic sliding contact. For nano-sized particles, the interfacial area is large relative to particle volume, and the particles are generally well bonded to the matrix due to their uniform dispersion and complete embedding. This results in fewer and smaller microdefects, and any cracks that do initiate are effectively arrested by the fine-grained structure. For micron-sized particles, particularly the 2 μm particles, the particle-matrix interfaces are more prone to debonding due to stress concentration and incomplete embedding. These interfacial gaps act as microdefects that promote crack propagation and subsequent particle pull-out. Once pulled out, the vacant sites further increase the defect density on the worn surface, accelerating material removal in subsequent sliding passes. Thirdly, the cross-sectional profiles of the wear tracks provide direct evidence of the wear mechanism transition. The C0 coating exhibits the widest and deepest wear track, with a large cross-sectional area removed, indicative of severe adhesive wear and extensive plastic deformation. The C-50 nm and C-1 μm coatings show significantly narrower and shallower wear tracks, with the cross-sectional areas substantially reduced compared to the C0 coating. This reduction in wear volume is attributed to the combined effect of higher hardness and the transition to a mixed wear mechanism. The C-2 μm coating shows a moderate reduction in cross-sectional wear area, which is consistent with its intermediate wear resistance.

The overall transformation from adhesive wear in the C0 coating to mixed adhesive-abrasive wear in the composite coatings is governed by particle size. Nano-sized Y_2_O_3_ particles provide the most effective reinforcement through firm embedding, fine debris formation, and minimal microdefect generation, resulting in the best wear resistance. Micron-sized particles, while still beneficial compared to the unreinforced coating, introduce more severe abrasive wear due to larger debris size and more pronounced particle pull-out, resulting in intermediate wear performance.

## 4. Conclusions

The influence of Y_2_O_3_ particle size on the microstructure, corrosion resistance, microhardness, and wear resistance of the electrodeposited Ni-W-Y_2_O_3_ composite coatings was systematically investigated. Main conclusions are reported in the following:

The incorporation of both micro and nano-sized Y_2_O_3_ particles notably influences the microstructure of Ni-W-Y_2_O_3_ composite coatings. Micro-sized particles effectively mitigate the nodular structures on the coating surface, thereby promoting the formation of a denser and more uniform surface morphology. Nano-sized Y_2_O_3_ particles further enhance the uniformity of the composite coatings, resulting in a more homogeneous distribution of the reinforcement within the Ni-W matrix.

In a 10 wt.% H_2_SO_4_ acid corrosive medium, the C-50 nm coating exhibits superior corrosion resistance compared to C-1 μm and C-2 μm coatings, which show similar corrosion resistance to the C0 alloy coating. Conversely, in a 3.5 wt.% NaCl neutral corrosive medium, the C-50 nm composite coating demonstrates enhanced corrosion resistance, whereas the corrosion resistance of the C-1 μm and C-2 μm composite coatings is notably inferior. The superior corrosion and wear resistance of the nano Y_2_O_3_ reinforced Ni W composite coatings make them promising candidates for protective applications in marine environments, including offshore platform components and shipboard equipment, where combined resistance to chloride-induced corrosion and mechanical wear is essential.

The microhardness of Ni-W-Y_2_O_3_ composite coatings is significantly improved by the addition of Y_2_O_3_ particles. Specifically, the microhardness of the C0 coating is measured at 540.2 HV. The C-50 nm coating achieves a microhardness of 732.5 HV, while the C-1 μm and C-2 μm coatings show microhardness values of 641.0 HV and 629.5 HV, respectively.

The incorporation of micro/nano Y_2_O_3_ particles enhances the wear resistance of Ni-W-Y_2_O_3_ composite coatings. The wear tracks of Ni-W alloy coatings exhibit extensive plastic deformation and adhesive wear, with the wear mechanism predominantly governed by adhesive wear. After the incorporation of Y_2_O_3_ particles, the wear tracks in the Ni-W-Y_2_O_3_ composite coatings become narrower and shallower. The wear mechanisms shift to a combination of adhesive and abrasive wear, indicating improved wear resistance.

Future work could explore the synergistic effects of incorporating multiple particle sizes or developing functionally graded coatings to further optimize the balance between corrosion resistance, wear resistance, and processability for specific engineering requirements.

## Figures and Tables

**Table 1:** Electrodeposition conditions and electrolyte compositions.

Electrolyte Composition	Concentration (g/L)	Electrodeposition Condition
NiSO_4_·6H_2_O	40	Current density: 2 A/dm^2^
NiCl_4_·6H_2_O	12	pH: 8.0 ± 0.2
Na_3_C_6_H_5_O_7_·2H_2_O	158	Magnetic agitation: 300 rpm
Na_2_WO_4_·2H_2_O	66	Interelectrode distance: 3 cm
NH_4_Cl	28	Temperature: 70 ± 1°C
C_12_H_25_NaO_4_S (SDS)	0.5	Duration: 60 min
Saccharin	1	Y_2_O_3_ particle size: 50 nm, 1 μm, 2 μm
Y_2_O_3_	10	
Anode: pure nickel sheet (15 cm × 15 cm)		
Cathode: stainless-steel plate (15 cm × 15 cm)		

**Table 2:** Elemental mass ratios of Ni-W-Y_2_O_3_ composite coatings reinforced by Y_2_O_3_ particles of different sizes.

Sample Code	Mass Ratios of Elements (wt.%)			
	Ni	W	O	Y
C0	73.68	26.32	—	—
C-50 nm	65.93	31.09	1.33	1.65
C-1 μm	67.91	28.63	1.54	1.93
C-2 μm	62.08	30.23	2.24	5.45

**Table 3:** Electrochemical corrosion data of the different Ni-W-Y_2_O_3_ composite coatings in 10 wt.% H_2_SO_4_ solution.

Sample Code	E_corr_ (vs. SCE, mV)	I_corr_ (μA/cm^2^)
C0	−315	4.85
C-50 nm	−287	3.72
C-1 μm	−307	4.68
C-2 μm	−318	5.32

**Table 4:** Electrochemical equivalent circuit parameters for the different Ni-W-Y_2_O_3_ composite coatings in 10 wt.% H_2_SO_4_ solution.

Sample Code	R_s_ (Ω cm^2^)	R_f_ (Ω cm^2^)	R_ct_ (Ω cm^2^)	CPE_f_ (Ω cm^2^)	CPE_ct_ (Ω cm^2^)
C0	6.3	490.3	189.2	1.1 × 10^−4^	1.3 × 10^−4^
C-50 nm	5.9	732.2	1342.0	4.4 × 10^−4^	3.8 × 10^−2^
C-1 μm	5.5	669.7	654.1	2.4 × 10^−4^	4.7 × 10^−2^
C-2 μm	6.3	580.9	207.4	3.1 × 10^−4^	1.7 × 10^−1^

**Table 5:** Electrochemical corrosion data of the different Ni-W-Y_2_O_3_ composite coatings in 3.5 wt.% NaCl neutral solution.

Sample Code	E_corr_ (vs. SCE, mV)	I_corr_ (μA/cm^2^)
C0	−398	4.35
C-50 nm	−382	3.53
C-1 μm	−443	7.68
C-2 μm	−437	7.74

**Table 6:** Electrochemical equivalent circuit parameters for the different Ni-W-Y_2_O_3_ composite coatings in 3.5 wt.% NaCl solution.

Sample Code	R_s_ (Ω cm^2^)	R_c_ (Ω cm^2^)	R_ct_ (Ω cm^2^)	CPE_c_ (Ω cm^2^)	CPE_dl_ (Ω cm^2^)
C0	32.33	1.23 × 10^4^	1.19 × 10^4^	1.79 × 10^−5^	1.28 × 10^−4^
C-50 nm	38.00	1.38 × 10^4^	1.36 × 10^4^	1.67 × 10^−5^	7.97 × 10^−5^
C-1 μm	33.44	1.15 × 10^4^	1.16 × 10^4^	2.86 × 10^−5^	1.63 × 10^−4^
C-2 μm	35.95	0.53 × 10^4^	1.09 × 10^4^	3.54 × 10^−5^	2.63 × 10^−4^

## Data Availability

The original contributions presented in this study are included in the article. Further inquiries can be directed to the corresponding author.
